# “3D Counterpart Analysis”: A Novel Method for Enlow’s Counterpart Analysis on CBCT

**DOI:** 10.3390/diagnostics12102513

**Published:** 2022-10-17

**Authors:** Michele D’Attilio, Antonino Peluso, Giulia Falone, Rossana Pipitone, Francesco Moscagiuri, Francesco Caroccia

**Affiliations:** Department of Innovative Technologies in Medicine & Dentistry, University “G. d’Annunzio” Chieti-Pescara, 66100 Chieti, Italy

**Keywords:** anthropometry, cephalometry, cone-beam computed tomography, dental diagnosis, radiography, orthodontics

## Abstract

The aim of this study was to propose a novel 3D Enlow’s counterpart analysis traced on cone-beam computed tomography (CBCT) images. Eighteen CBCT images of skeletal Class I (ANB = 2° ± 2°) subjects (12 males and 6 females, aged from 9 to 19 years) with no history of previous orthodontic treatment were selected. For each subject, a 2D Enlow’s counterpart analysis was performed on lateral cephalograms extracted from the CBCT images. The following structures were identified: mandibular ramus, middle cranial floor, maxillary skeletal arch, mandibular skeletal arch, maxillary dento-alveolar arch, mandibular dento-alveolar arch. The differences between each part and its relative counterpart obtained from the 2D analysis were than compared with those obtained from a 3D analysis traced on the CBCT images. A Student’s *t*-test did not show any statistical significant difference between the 2D and 3D measurements. The landmarks proposed by this study identified the cranio-facial structures on the 3D images in a way that could be superimposed on those described by Enlow in his analysis performed on 2D lateral cephalograms.

## 1. Introduction

The study of lateral cephalograms is absolutely common in daily practice among orthodontists and oro-maxillofacial surgeons and it is fundamental for a correct diagnosis as well as for drafting a correct treatment plan. Lateral radiographs can be used to evaluate dentofacial and craniofacial proportions and even growth changes. However, a cephalometric study traced on 2D images has intrinsic limitations due to magnification, geometric distortion, and above all, superimpositions of anatomical structures. Three-dimensional (3D) diagnostic imaging could overcome these limitations of the 2D imaging [1] even with a low dose of radiation absorption for the patient [2,3]. Three-dimensional imaging is now widespread in orthodontics, with devices ranging from intraoral scanners to radiology systems such as cone-beam computet tomography (CBCT) machines. Three-dimensional imaging has several advantages as compared with medical a CT: lower radiation dose exposure, compact dimension that allows devices to be installed in a common dental office, and no requirement for a patient to be lying down for the scan.

The need to overcome the limits of the 2D cephalometric analysis has led clinicians to develop different CBCT-based methods of 3D cephalometric analysis in the last few years. Swennen and Schutyser [4] described the potential of the CBCT 3D cephalometry. Gateno et al. [5] proposed a new 3D cephalometric analysis for measuring the size, shape, position and orientation of different facial units and introduced a novel method to measure asymmetries. Farronato et al. [1] proposed a 10-point 3D analysis of CBCT images which showed that the small number of points to be selected drastically reduce human error as compared with the 2D Steiner cephalometry method. Furthermore, Farronato et al. [1] concluded that the information provided by the graphical representation facilitated orthodontic diagnosis making it more reliable and repeatable. Perrotti et al. [6] proposed a novel 3D cephalometric analysis with relative normal values of reference.

Some authors have also proposed some non-radiographic 3D methods. Juerchott et al. investigated the reliability of 3D cephalometric landmarks traced on magnetic resonance imaging (MRI) records [7]. Catillo et al. demonstrated that jaws relation and incisors orientation could be predicted from 3D photogrammetry measurements [8].

However, two recent systematic reviews by Pittayapat et al. [9] and Smektała et al. [10] concluded that 3D cephalometry still had limited evidence of diagnostic efficiency. Furthermore, the use of CBCT images in orthodontics is not wide spread, due to the greater exposure to radiation as compared with teleradiographs, despite the existence of low-dose image acquisition protocols. Indeed, some studies have also proposed cephalometric analysis obtained with a reduced field of view (FOV) in order to combine the advantages of three-dimensional imaging with a further reduction in radiation exposure. The reduced FOV extending from the Frankfurt plane to the lowest point on the chin symphysis (Me) as compared with a large FOV demonstrated the reliability of the measurements performed while reducing radiation exposure [11,12].

Several cephalometric methods have been published in the literature to analyze the cranio-mandibular structures on lateral cephalograms and, currently, even some automated landmark detection and tracing systems have been proposed [13].

Donald H. Enlow in his “counterpart analysis” [14] proposed a method to correlate vertical and horizontal dimensions that led to a balanced or unbalanced model of the face. The counterpart analysis examined the shape, location, and development of each structural component within the framework of the cranio-mandible-cervical whole and comparing each structure with the corresponding counterpart using the “millimeter difference method”, in which the condition with from 0 to 2.5 mm difference between part and counterpart was considered to be harmonic and ideal.

In an era of increasingly digital orthodontics and three-dimensional orthodontic analysis and design, it is possible to overcome the difficulties of a two-dimensional cephalometric analysis and review the old cephalometric notions in light of modern technologies. Therefore, the aim is to increase the precision of the analysis without overlapping of anatomical structures or points constructed radiographically.

The purpose of this study was to present a new 3D Enlow’s horizontal counterpart analysis that used anatomical points traced on CBCT images, in order to identify the different horizontal dimensions of Enlow’s 2D counterpart analysis.

## 2. Materials and Methods

Eighteen CBCT images of skeletal Class I (ANB = 2° ± 2°) subjects (12 males and 6 females, aged from 9 to 19 years) with no history of previous orthodontic treatment were selected from the archives of the Unit of Orthodontics, Department of Innovative Technologies in Medicine and Dentistry, University of Chieti, Chieti, Italy. The radiographic images were obtained by using a low dose CBCT machine (Vatechlpax 3D PCH-6500, Fort Lee, NJ, USA) and processed using the Ez3D Plus Software (Vatech, Global Fort Lee, NJ, USA). The scanning procedure has been previously described by Moscagiuri et al. [15]. For each subject, lateral cephalograms were extracted from a CBCT image and evaluated with Enlow’s counterpart analysis [16,17] by using the OrisCeph3 (OrisLine; Elite Computer Italia S.r.l., Milano, Italy) software and the CBCT scans were evaluated with a 3D cephalometric analysis by using the Materialise Mimics software (Materialise NV, Leuven, Belgium). Enlow’s horizontal counterpart analysis were performed on both lateral cephalograms and CBCT images.

### 2.1. 2D Horizontal Counterpart Analysis

**Figure 1:**
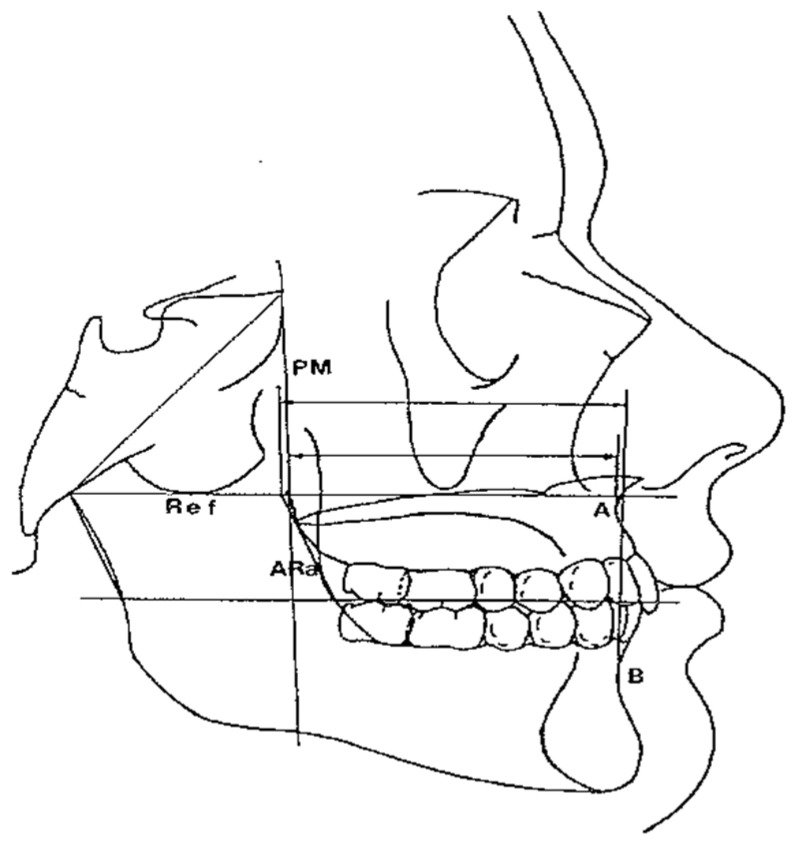
Maxillary/mandibular skeletal arches at A/B.

**Figure 2:**
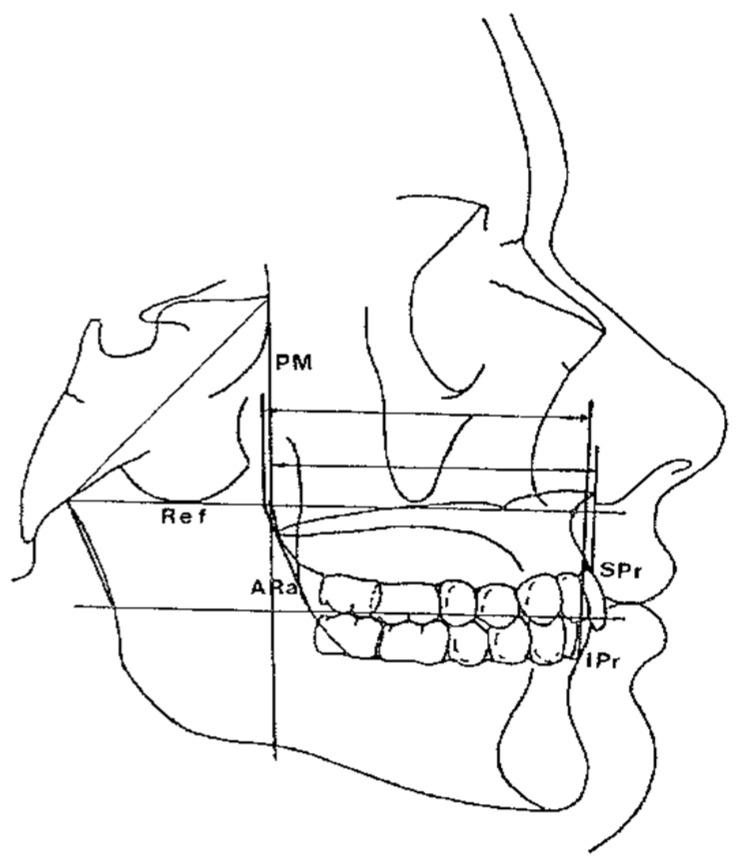
Maxillary/mandibular dento-alveolar arches at SPr/IPr.

**Figure 3:**
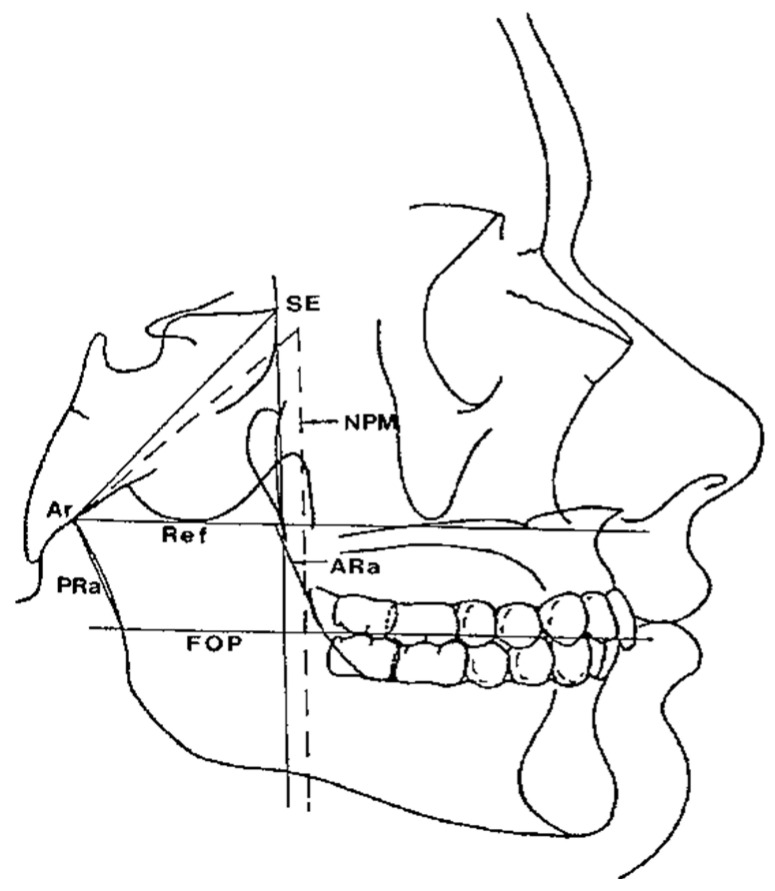
Ramus/MCF horizontal skeletal dimension.

Enlow’s Counterpart Analysis was performed on lateral cephalograms identifying the landmarks described in Table 1:

The horizontal counterpart analysis measures the following counterparts:

- Maxillary and Mandibular skeletal arches (Figure 1): this parameter compares the lengths of the maxillary and mandibular skeletal arches. The maxillary skeletal arch was measured by the distance between A point and PM, in parallel with Ref. The mandibular skeletal arch was measured by the distance between the orthogonal projection of B point on Ref and ARa, at its intersection point with Ref.
- Maxillary and Mandibular dento-alveolar arches (Figure 2): this parameter compares the lengths of maxillary and mandibular dento-alveolar arches. The maxillary dental arch was measured by the distance between PM and SPr, in parallel with Ref. The mandibular dental arch was measured by the distance between the orthogonal projection of IPr on Ref and Ara, at its intersection point with Ref.
- Middle Cranial Floor (MCF) and Mandibular ramus (Figure 3): this parameter compares the horizontal dimension of the middle cranial floor (Ar to neutral PM) and the width of the ramus (Ar to ARa). These parameters were measured along the Ref.

### 2.2. 3D Horizontal Counterpart Analysis

**Figure 4:**
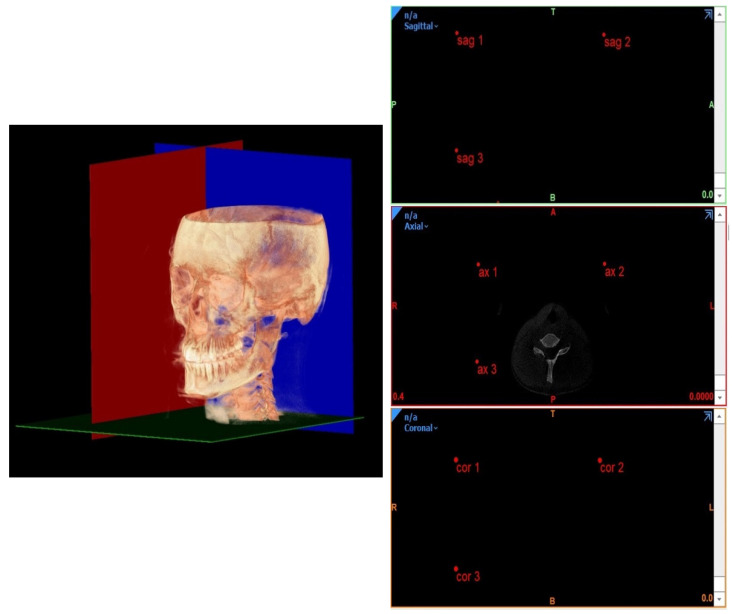
CBCT orientation according to three reference planes identified by selecting 3 points on the most external slice of the CBCT scan, respectively, for every single view (coronal, axial, and sagittal).

Before tracing, the CBCT images were orientated according to three reference planes identified by selecting three points on the most external slice of the CBCT scan, respectively, for every single view (coronal, axial, and sagittal), as previously done by Perrotti et al. [6] (Figure 4).

Planes identified during the 3D cephalometric analysis were traced according to the following geometric rules of planes construction:

- Planes passing through 3 points;
- Planes passing through 2 points normal to a plane;
- Planes passing through 1 point parallel to a plane.

By using these planes, the distances between a point and a plane were automatically measured by the software with the function “measure and analyze” from the software’s tool menu.

The landmarks coordination used in the 3D analysis are explained in Table 2.

**Figure 5:**
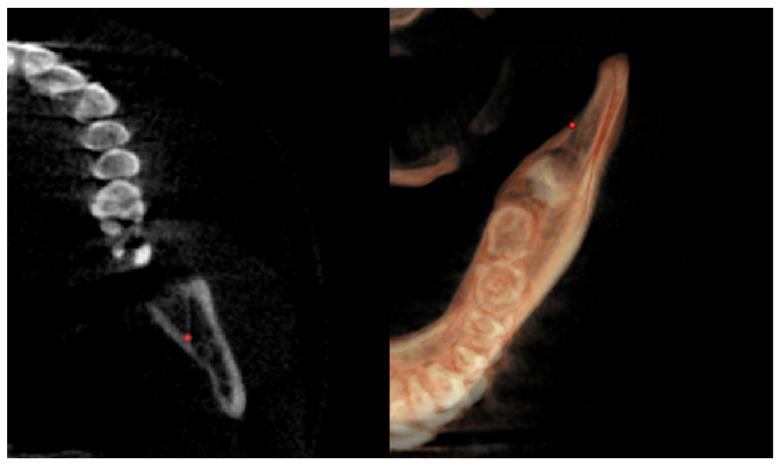
CBCT images showing the anatomical lingual tuberosity.

The anatomical lingual tuberosity (Figure 5) was located on the 3D according to the Enlow’s references in the book “*Essentials of Facial Growth*” [14] and anatomical references from the books “*Gray’s Anatomy for Students*” [17] and “*Sicher’s Oral Anatomy*” [18]. As Enlow explained, the lingual tuberosity represents “the effective boundary between the two basic part of the mandible: the ramus and corpus. The inaccessibility of the lingual tuberosity for routine cephalometric study is a great loss” [14]. This is due to the overlapping by the ramus in the lateral headfilm because the lingual tuberosity lies toward the midline from the ramus. The book “*Gray’s anatomy for Students*” defined it as the “retromolar triangle” the area posterior to the last molar [17]; regarding the “*Sicher’s Oral Anatomy*”, this triangle has the “medial and the lateral margins continuous with the alveolar bone, respectively, on the buccal and the lingual side, of the last molar” [18]. Based on these anatomic references, the position of the lingual tuberosity has been defined and the two lingual tuberosity points define a plane that separates the mandible into two portions: the anterior representing the mandibular corpus and the posterior representing the mandibular ramus.

On the 3D, the structures previously described were evaluated with the following method:

**Maxillary and Mandibular skeletal arches**

**Figure 6:**
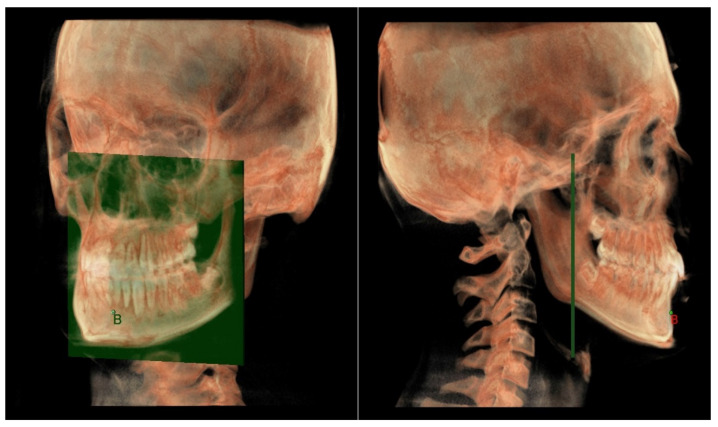
Maxillary skeletal arch in 3D analysis.

**Figure 7:**
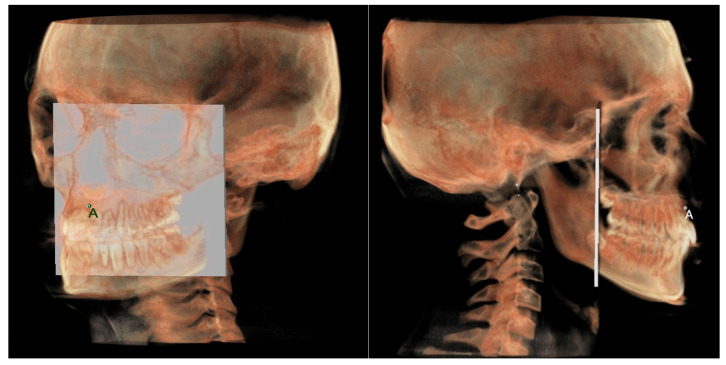
Mandibular skeletal arch in 3D analysis.

○ The maxillary skeletal arch (Figure 6) was measured from point A to a plane passing through the PNS and parallel to the coronal plane.
○ The mandibular skeletal arch (Figure 7) was measured from the point B to a plane passing through the two lingual tuberosity and normal to the axial plane

**Maxillary and mandibular dento-alveolar arches**

**Figure 8:**
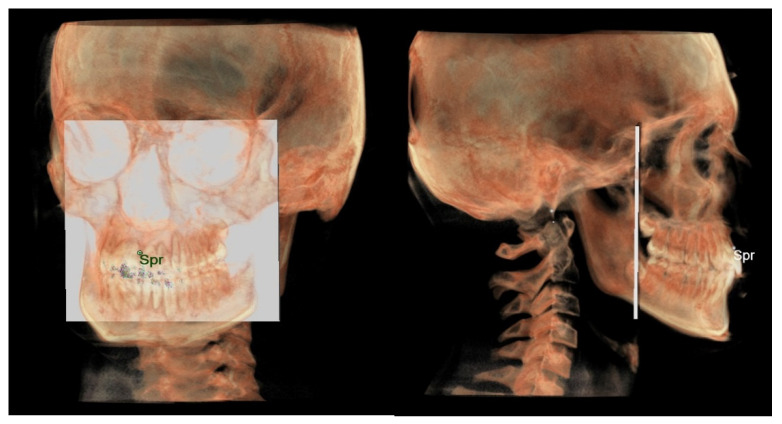
Maxillary dento-alveolar arch in 3D analysis.

**Figure 9:**
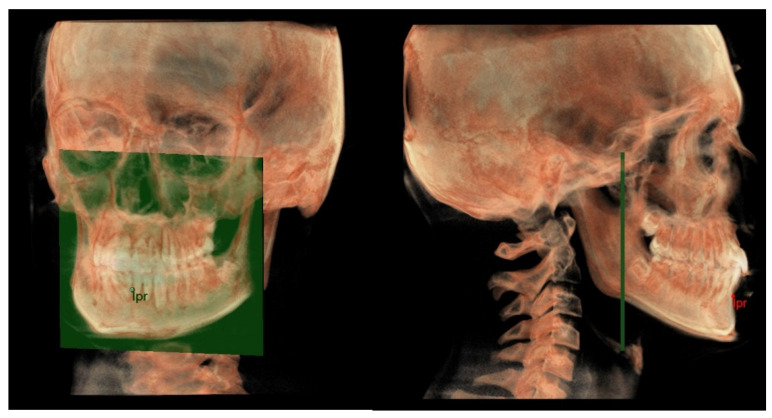
Mandibular dento-alveolar arch in 3D analysis.

○ The maxillary dento-alveolar arch (Figure 8) was measured from the point SPr to a plane passing through the PNS and parallel to the coronal plane.
○ The mandibular dento-alveolar arch (Figure 9) was measured from the point IPr to a plane passing through the two lingual tuberosity and normal to the axial plane.

**Middle cranial floor and mandibular ramus**

**Figure 10:**
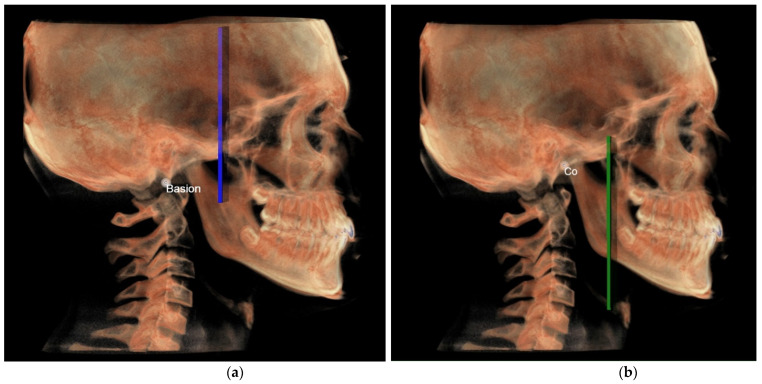
(**a**) Middle cranial fossa (MCF) in 3D analysis; (**b**) ramus in 3D analysis.

○ The middle cranial floor (Figure 10) was determined by the distance from Ba to a plane passing through the two anterior clinoid processes and normal to the axial plane. The anterior clinoid processes were chosen as they represented the most posterior point of the anterior cranial floor and the most anterior point of the middle cranial floor.
○ The mandibular ramus was measured from the right and left condylion to a plane passing through the right and left lingual tuberosity and normal to the axial plane (Figure 10).

### 2.3. Statistical Analysis

**Figure 11:**
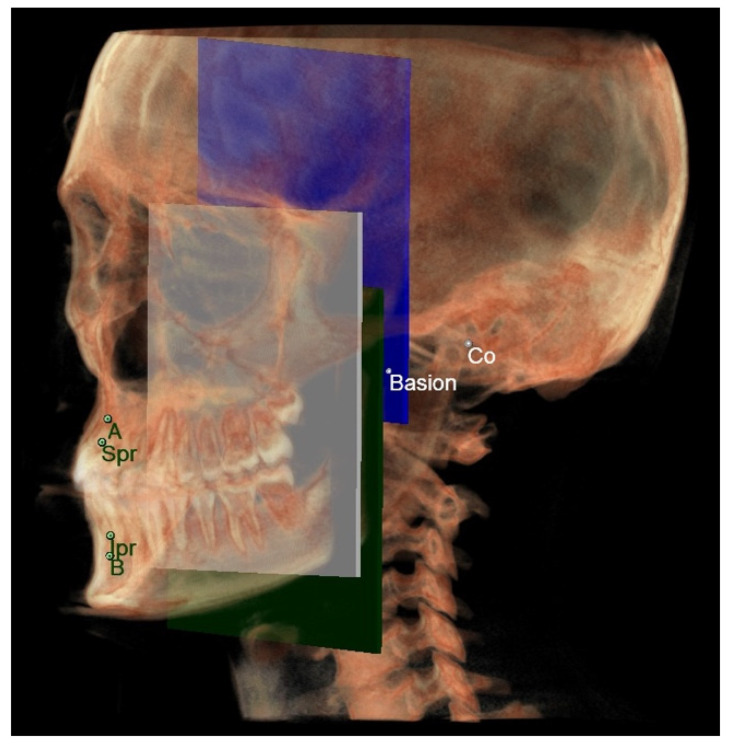
All the planes and points of the 3D horizontal analysis.

To validate the proposed landmarks for the “3D horizontal counterpart analysis” (Figure 11), the average measures obtained from the discrepancy between each part and its counterpart on the 2D and 3D were compared to confirm the null hypothesis of the absence of statistically significant differences between the analyzing method used on the 2D and the 3D.

To evaluate the discrepancy between the middle cranial fossa and the mandibular ramus on the 3D, the values obtained from the measurement of the right ramus were conventionally chosen.

Statistical analysis was performed using the Prism-GraphPad software (Graphpad software, LLC, San Diego, CA, USA). The Kolmogorov–Smirnov normality test was applied for each variable to check whether data were normally distributed. Since data were normally distributed, the paired Student’s *t*-test was performed. The level of statistical significance was 0.05.

## 3. Results

A total of eighteen cephalograms were extracted from the CBCT images and evaluated. The 2D Enlow’s counterpart analysis and 3D analysis were traced on lateral cephalograms extracted from CBCT and on the CBCT images, respectively.

### Horizontal Counterpart Analysis

The discrepancy values between the three variables according to Enlow’s counterpart analysis were calculated. Data were obtained by subtraction between parties and counterparties according to Enlow’s analysis. Table 3 shows means and standard deviations of each discrepancy, first in 2D and then in 3D: maxillary and mandibular skeletal arch, maxillary and mandibular dento-alveolar arch, middle cranial floor and mandibular ramus.

Once the Kolmogorov–Smirnov test was performed and verified that the sample was normally distributed, the paired Student’s *t*-test was performed.

The gap between the discrepancy values of each variable was small and no statistically significant difference was highlighted by the unpaired Student’s *t*-test (*p* > 0.05), confirming the null hypothesis and proving the validity of the proposed new method for 3D horizontal counterpart analysis.

## 4. Discussion

The aim of this study was to present a 3D cephalometric analysis based on the craniofacial growth theories and the related cephalometric analysis described by D.H. Enlow [14]. The idea of performing a 3D cephalometric analysis stems from the possibility for modern orthodontists to obtain three-dimensional images of maxillofacial structures through the use of technologies and protocols that significantly reduce the exposure of radiation for the patient such as low dose CBCT [2,3,19]. Given the lack of standardized protocols in 3D cephalometry, this preliminary study aims to provide an easily reproducible method of analysis that can contribute to a 3D orthodontic diagnosis.

One of the main advantages of using CBCT images to run a cephalometric analysis is represented by the possibility of avoiding splits or superimpositions of the images and to be able to bilaterally evaluate the symmetrical structures. For instance, 3D imaging can e used to identify the anatomical point describing the lingual tuberosity considered to be the connection point between the body and the mandibular ramus, an area that is not visible in the common teleradiographs as it is covered by the mandibular branch.

Moreover, our cephalometric analysis was performed solely by anatomical points, without radiographically constructed points, which are the most frequently mistaken.

All the measurements of the 3D analysis of the horizontal counterparts were obtained as a distance between a point and a plane. This method represents a different approach than those described in the literature where usually the length of maxillofacial structures is measured as a distance between two or three points. In our analysis, the ability to use plans provides us with several benefits. The first is the possibility of dividing the skull into different portions. Indeed, the presence of planes allow us to divide the mandible into mandibular body and mandibular ramus and, consequently, measure these two portions separately; it also allows us to identify a repeatable division zone at the level of the skull base that separates the middle cranial floor from the anterior cranial floor. The second advantage of using planes is the possibility of standardizing the measurement between points that do not lie on the same plane, for instance, the evaluation of the mandibular ramus where the condylion and the lingual tuberosity points are located at different heights. Finally, the method proposed in this study to construct the reference planes seems to be easier and less operator dependent as compared with other methods described in the literature [1,5,20,21,22,23]. Selecting three points outside the skull to orientate the images could also avoid the displacement of these landmarks due to local bone remodeling [6]. Furthermore, the lower number of points to select and the greater ease in their identification makes the 3D analysis a more accurate analysis with a lower risk of operator-dependent errors.

Moreover, the transition from 2D cephalometry to 3D determines a large number of data that can appear complex to clinicians. One of the approaches described to prevent this “information overload” is to create 3D average hard and soft tissue models, but the attempts to generate accurate average 3D skull models are still a challenge because of the complex anatomy [24]. This problem can be overcome with our analysis since each subject is compared with himself and not with average values from the population, reducing the amount of information to combine for the diagnosis.

The results obtained from the statistical analysis showed no statistically significant differences between the 2D and 3D measurements obtained by the difference between the part and its relative counterpart, as described by D.H. Enlow [16]. The landmarks proposed by this study identified cranio-facial structures on 3D images in a way that could be superimposed on those described by Enlow in his analysis performed on 2D lateral cephalograms. Due to the anatomical positions of the points identified in this 3D analysis, a large FOV is necessary and a reduced FOV cannot be used. With current radiographic acquisition protocols, this analysis may not be used in routinary orthodontic diagnosis due to radiation exposure. It could be useful for more complex cases, situations were teleradiographs could show superimpositions (i.e., skeletal asymmetries), patients who will undergo orthognathic surgery, or cases where CBCT is already prescribed for other clinical reasons (i.e., dental inclusions).

Another limitation is that the sample investigated in our study is composed exclusively using skeletal Class I subjects. Future investigations should verify the reproducibility of the points proposed in this analysis, also in different sagittal and vertical skeletal relationships.

Future investigations could be performed on the basis of this preliminary study to identify all the landmarks of the neutral track and vertical counterpart analysis proposed by D.H. Enlow and to reach a complete 3D counterpart analysis.

## 5. Conclusions

The landmarks proposed by this study to identify on 3D images the mandibular ramus, middle cranial floor, maxillary skeletal arch, mandibular skeletal arch, maxillary dento-alveolar arch, and mandibular dento-alveolar arch are valid and superimposable on those described by Enlow in his analysis performed on 2D lateral cephalograms.

## Figures and Tables

**Table 1:** Landmarks and lines identified in the 2D simplified Enlow’s analysis.

Landmarks	
Go	Gonion: Geometric construction point given by the intersection of two lines, one passing from Me to the lower most point of the mandibular corpus, the other passing from Ar to the posterior most point of mandibular ramus
Ar	Articulare: Point of intersection between the posterior margin of the ramus and the outer margin of the cranial base
Me	Menton: Most inferior point of the mandibular symphysis
SE	Sphenoethmoidal junction: Intersection of the averaged image of the right and left shadows of the great wings of the sphenoid with the floor of the anterior cranial fossae
PTM	Lowest point in the contour of the pterygomaxillary fissure formed anteriorly by the retromolar tuberosity of the maxilla and posteriorly by the anterior curve of the pterigoid process of the sphenoid bone
Poc	Posterior occlusal contact: Most supero-distal contact point of the first molars.
Aoc	Anterior occlusal contact: Most mesial contact point of the first premolars or first deciduous molars.
A	Most concave point of the anterior maxilla
SPr	Superior Prostion: Occlusal limit of bone papillae of the maxillary alveolar bone
IPr	Inferior Prostion, occlusal limit of bone papillae of the mandibular alveolar bone
B	Most concave point of the mandibular symphysis
LT	Lingual tuberosity: Point of intersection between the FOP and the anterior border of mandibular ramus
**Lines**	
FOP	Functional occlusal plane, Poc–Aoc
PM	Pterygo-mandibular plane, SE-PTM
MCF	Middle cranial floor plane, Ar-SE
PRa	Posterior ramus alignment: Distance from point Ar to the point where the FOP intersects the posterior margin of the mandibular ramus
ARa	Anterior ramus alignment: Line parallel to the PRa from LT to the ref line
Ref	Reference plane: Line parallel to the FOP passing through point Ar
MR	Mandibular ramus: Ar-Go

**Table 2:** Landmarks identified in the 3D analysis.

Point	X (Left to Right) Sagittal View	Y (Superior to Inferior) Axial View	Z (Posterior to Anterior) Coronal View
Basion (Ba)	/	Most anterior point of foramen magnum	/
Right condylion (rCo)	Most posterior point of the mandibular condyle, right side	Most posterior point of mandibular condyle, right side	Most posterior point of mandibular condyle, right side
Left condylion (lCo)	Most posterior point of mandibular condyle, left side	Most posterior point of mandibular condyle, left side	Most posterior point of mandibular condyle, left side
Right lingual tuberosity (rLT)	/	The most posterior point (apex) of the retromandibular triangle, right side (Figure 5)	/
Left lingual tuberosity (lLT)	/	The most posterior point (apex) of the retromandibular triangle, left side (Figure 5)	/
A	Most concave point on the midline of anterior maxilla	/	/
B	Most concave point on the midline of mandibular symphysis	/	/
SPr	Most anterior point on the maxillary alveolar process between the central incisor	Most anterior point on the maxillary alveolar process between the central incisor	Most anterior point on the maxillary alveolar process between the central incisor
IPr	Most anterior point on the mandibular alveolar process between the central incisor	Most anterior point on the mandibular alveolar process between the central incisor	Most anterior point on the mandibular alveolar process between the central incisor
Posterior nasal spine (PNS)	Most posterior point of the hard palate	Most posterior point of the hard palate	Most posterior point of the hard palate
Right anterior clinoid process	Most posterior point of the anterior clinoid process, right side	Most posterior point of the anterior clinoid process, right side	Most posterior point of the anterior clinoid process, right side
Left anterior clinoid process	Most posterior point of the anterior clinoid process, left side	Most posterior point of the anterior clinoid process, left side	Most posterior point of the anterior clinoid process, left side

**Table 3:** Comparing discrepancies of the horizontal counterpart analysis between 2D and 3D parameters.

Index	2D (Mean ± SD)	3D (Mean ± SD)	*p* Value ^a^
Middle cranial floor-mandibular ramus Maxillary-mandibular skeletal arches Maxillary-mandibular dento-alveolar arches	0.15 ± 0.12 cm	0.17 ± 0.09 cm	0.91
	−0.52 ± 0.25 cm	−0.54 ± 0.33 cm	0.89
	−0.29 ± 0.14 cm	−0.47 ± 0.13 cm	0.18

Legend: SD = standard deviation. ^a^ Paired Student’s *t*-test, level of significance was set at *p* < 0.05.

## Data Availability

The data presented in this study are available on request from the corresponding author.
